# Examining Black Birthing People’s Experiences with Racism, Discrimination, and Contextualized Stress and Their Perspectives on Racial Concordance with Prenatal Providers

**DOI:** 10.1089/heq.2023.0266

**Published:** 2024-09-12

**Authors:** E. Nicole Teal, Aryana Daye, Sarah C. Haight, M. Kathryn Menard, Karen Sheffield-Abdullah

**Affiliations:** ^1^ Division of Maternal-Fetal Medicine, Department of Obstetrics, Gynecology & Reproductive Sciences, University of California, San Diego, San Diego, California, USA.; ^2^ College of Arts and Sciences, University of North Carolina at Chapel Hill, Chapel Hill, North Carolina, USA.; ^3^ Department of Epidemiology, University of North Carolina at Chapel Hill, Chapel Hill, North Carolina, USA.; ^4^ School of Nursing, University of North Carolina at Chapel Hill, Chapel Hill, North Carolina, USA.

**Keywords:** racial concordance, prenatal care, obstetrics, black or African American, racism, discrimination, contextualized stress, continuity of care, patient–provider interaction

## Abstract

**Introduction:**

We examine Black birthing people’s experiences with racism, discrimination, and contextualized stress and whether those experiences are associated with preference for racially concordant prenatal care providers.

**Methods:**

This cross-sectional study is the quantitative component of a larger, mixed-methods study. Data were from initial (August–October 2021) and follow-up (December 2022–January 2023) surveys among self-identified Black and/or African American birthing people who delivered a baby at a university system between 2019 and 2021 and were at least 18 years old. Respondents were 3–32 months postdelivery at the initial survey, which collected data on demographics and the Perceived Racism (ranges 0–430), Perceived Discrimination (ranges 0–36), and Jackson, Hogue, Phillips Contextualized Stress Measure (ranges 0–355) scales. The follow-up survey assessed views on racial and gender concordance and continuity with prenatal providers. Pearson correlation coefficients assessed relationships between scale scores and agreement that racial concordance is important and preferable. Poisson regression assessed whether a top quartile score on scales was related to importance of and preference for racial concordance with providers.

**Results:**

Participants (*n* = 200) scored medians of 99.5 on the racism scale, 33 on the discrimination scale, and 177 on the contextualized stress scale. Of follow-up survey participants (*n* = 69), 78.3% agreed they would choose a racially concordant prenatal provider if possible (*n* = 54) and 42.0% agreed that racial concordance with their provider was important (*n* = 29). Scoring higher on discrimination and contextualized stress scales was positively correlated with agreeing that racial concordance was important. Regression analyses showed no significant associations between scale scores and agreeing that racial concordance with one’s prenatal provider is important or preferable.

**Conclusion:**

Black birthing people experience high levels of racism, discrimination, and contextualized stress. The overwhelming majority would choose racial concordance with their prenatal provider if possible.

## Introduction

Black birthing people in the United States have a greater than threefold increased risk of pregnancy-related mortality and twofold increased risk of maternal morbidity compared with White birthing people.^1,2^ There is growing attention to reducing these disturbing inequities through policy change, hospital quality improvement initiatives, prenatal care revamping, and reinforcement of preconception and postpartum care.^3^ Alternative models of prenatal care, including group prenatal care and the Pregnancy Medical Home, have shown some promise in reducing racial disparities in low birthweight and prematurity in small studies, but whether results are replicable remains insufficiently studied.^4–7^ Overall, there are minimal available data on ways to reduce disparities in pregnancy outcomes through prenatal care provision. Finding meaningful and sustainable strategies to improve quality of prenatal care for racially marginalized communities is of paramount importance.

Critical Race Theory and its accompanying public health methodology, presented by Ford & Airhihenbuwa, can guide investigation into viable strategies for improving the quality of prenatal care for Black birthing people.^8,9^ First, race is a social construct and, as such, any observed racial disparities in pregnancy outcomes must be attributed to experiences of racism and discrimination. In support of this notion, a multitude of research has indicated that Black birthing people in the United States experience racism, discrimination, and cumulative stress over the life course and, in turn, an increased risk of adverse birth outcomes.^10–15^

To understand discrimination within health care settings and its impacts, we consider another principal of the public health critical race praxis: the “ordinariness of racism,” which posits that the absence of overtly “egregious” acts of racism does not indicate an absence of racism.^8,9^ Discrimination by providers, whether macro or micro, can adversely impact physical and mental health^16^ and inhibit marginalized populations from receiving care.^17^ This pattern remains consistent for utilization of prenatal care^18^; a 2019 study found that experiences of racial microaggressions were associated with delayed prenatal care, and patient skin tone modified this relationship.^19^

Racial concordance between providers and patients may be one strategy to lessen experiences of discrimination in the health care setting and, in turn, improve quality of care. This phenomenon has been explored in primary care where racial concordance is associated with higher patient ratings of trust, satisfaction, and intended adherence to treatment plans.^20,21^ Patients with racially concordant providers were more likely to undergo preventative care measures and less likely to delay seeking care than those with racially discordant providers.^22,23^ Emerging literature has begun to explore these patterns within prenatal care. In 2020, Greenwood et al. demonstrated that although newborn–physician racial concordance was associated with Black infant mortality reduction, there was no similar association with birthing person–physician racial concordance.^24^ Additional studies conducted in 2022 found that racial concordance with prenatal providers was valued by Black birthing people and improved pregnancy and birth experiences.^25,26^ Importantly, a prospective study by Adams et al. (2022) showed that White birthing people were more likely to have racial concordance with their obstetric provider than non-White birthing people, although there were no significant differences in perceptions of interpersonal processes of care.^27^ The authors concluded that measures of care quality in obstetrics need to be clarified to address racial and ethnic disparities in outcomes.^27^ A 2020 qualitative study by Altman et al. asked women of color to share practical ways health care providers and systems could improve their pregnancy experiences and deduced three key recommendations as follows: (1) increase racial concordance, (2) improve continuity of care, and (3) reduce discrimination.^28^ Despite these findings, to date, there is a paucity of literature examining how experiences of racism and stress among Black birthing people may influence their preference for racial concordance with prenatal providers.

Informed by existing qualitative data and research performed in primary care settings, this study explores the association between Black birthing people’s experiences of racism, discrimination, and contextualized stress and their views on racial concordance with prenatal providers.^29^ We hypothesize that higher levels of experienced racism, discrimination, and contextualized stress will be associated with stronger preference for racial concordance with one’s prenatal provider. Secondary aims are to assess whether experiences of discrimination, racism, and contextualized stress correlate with one another and whether demographic characteristics influence those experiences.

## Methods

This cross-sectional study is the quantitative component of a larger, mixed-methods study. Data stem from an initial survey administered from August 2021 to October 2021 and a follow-up survey administered from December 2022 to January 2023.

Participants were eligible for enrollment if they self-identified as Black/African American, were at least 18 years old, and had delivered a baby within our large university system between 2019 and 2021. Participant race was obtained from the electronic health record, but confirmed with self-report during the enrollment process. Non-Black individuals were excluded because our overarching goal was to illuminate strategies to improve outcomes for Black birthing people by centering their lived experiences. Non-English-speaking individuals were excluded given the scales used have not been validated in other languages.

We recruited eligible participants from a central data repository sourced from our large health care system. A recruitment email was sent to 3,237 individuals with links to an eligibility confirmation form, consent form, and the investigator’s contact information so that interested participants could contact the researcher via email, telephone, or text. An initial survey was administered to interested participants from August 2021 to October 2021; respondents were 3–32 months postpartum. The initial survey included a demographic questionnaire (Supplementary Appendix S1) and three self-reported questionnaires: the Perceived Racism Scale (racism scale), the Perceived Discrimination Scale (discrimination scale), and the Jackson, Hogue, Phillips Contextualized Stress Measure (contextualized stress scale).^29–31^ The estimated time to complete this survey was approximately 30 min. The survey automatically closed once 200 individuals completed it to mimic similar studies’ sample sizes while remaining within the study budget.^20,25–27^ Participants received a $15 gift card for completing the initial survey. While reviewing the study data, the research team determined it would be prudent to administer a follow-up survey assessing participants’ perceived importance of and preference for racial concordance, gender concordance, and continuity with prenatal providers. All 200 participants from the initial survey were invited to participate in the follow-up survey on racial concordance, gender concordance, and continuity of care with providers, administered from December 2022 to January 2023. Sixty-nine individuals completed the follow-up survey and were 26–48 months postpartum. No additional compensation was offered for the follow-up survey. All surveys were collected and managed using a secure electronic data capture tool, REDCap (Research Electronic Data Capture), hosted at the University of North Carolina at Chapel Hill.^32^ Study procedures and informed consent forms were reviewed and approved by the university’s Institutional Review Board for the Protection of Human Subjects.

### Measures

The Perceived Racism Scale is a multidimensional scale designed specifically to assess the experience of racism among African Americans.^30^ The 51-item scale includes questions regarding the frequency of exposure to racism at work, in academic settings, and in the public realm, including exposure to racist statements (e.g., “Blacks have gotten more economical and educational breaks than they deserve.”).^30^ Likert response options included the following: not applicable, almost never, several times a year, several times a month, several times a week, and several times a day with scores of 0–5, respectively, that were summed across the 51 items with a possible range of 0–430. In bivariate models, scores were made binary where scores in the top quartile (≥137.5) were considered heightened levels of perceived racism and scores <137.5 were considered the reference group.

The Perceived Discrimination Scale measures the frequency with which individuals feel others treat them unfairly on the basis of race, ethnicity, gender, age, religion, physical appearance, or sexual orientation.^31^ It includes multiple aspects of life such as school, work, and neighborhood and has been validated in the United States with people of diverse races, ages, education levels, and socioeconomic backgrounds.^33^ Its 20 items are divided into two subscales as follows: The Lifetime Discrimination Scale and the Daily Discrimination Scale.^31^ For the 11 Lifetime Discrimination items, 1 point is assigned to every item that has occurred one or more times. For the 9 Daily Discrimination items, Likert response options included never, rarely, sometimes, and often with scores of 1–4, respectively. Scores were summed across the 20 items with a possible range of 0–36. In bivariate models, scores were made binary where scores in the top quartile ( ≥42.0) were considered heightened levels of perceived discrimination and scores <42.0 were considered the reference group.

The Jackson, Hogue, Phillips Contextualized Stress Measure is a validated 71-item measure designed specifically to capture the authentic experiences of stress and stress mediators for African American women.^29^ It focuses on contextualized stress, inclusive of chronic exposure to racial and gendered stress. Its subscales include racism, burden, coping, personal history, and work stress.^29^ Likert responses options included strongly agree, agree, unsure, disagree, and strongly disagree with scores of 1–5, respectively, that were summed across the 71 items with a possible range of 0–355. In bivariate models, scores were made binary where scores in the top quartile ( ≥203.0) were considered heightened levels of contextualized stress and scores <203.0 were considered the reference group.

The follow-up survey included 6 statements: 3 about the importance of racial concordance, gender concordance, and continuity with one’s obstetric provider and 3 about whether the participant would choose racial concordance, gender concordance, and continuity with one’s obstetric provider *if given the opportunit*y. Five Likert response options included strongly disagree, disagree, neutral, agree, and strongly agree (Supplementary Appendix S1). Racial and gender concordance was defined for participants as having a shared racial or gender identity, respectively, with their provider. Responses were operationalized as binary variables where any indication of “agree” or “strongly agree” was considered to be perceived importance of or preference for racial concordance, gender concordance, and continuity with one’s obstetric provider.

Sociodemographic characteristics included the following: age (continuous), number of children (continuous), household size including partner (continuous), ethnicity (Hispanic, non-Hispanic, chose not to answer), marital status (married, unmarried), education (less than a high school diploma, high school diploma, some college, associate degree, bachelor’s degree, master’s degree, professional degree, and doctorate), and annual household income (<$20,000K; $20,000–$34,999; $35,000–$49,999; $50,000–$64,999; $65,000–$79,999; $80,000–$99,999; $100,000). For bivariate regression models, demographic characteristics were made binary: age ( ≥35 years old vs. <35 years old), marital status (unmarried vs. married), education level (<bachelor’s degree vs. ≥bachelor’s degree), and annual household income (<$50k per year vs. ≥$50K per year).

### Analysis

Descriptive statistics were calculated for participant characteristics of the initial and follow-up samples; the racism, discrimination, and contextualized stress scales; and the reported importance of and preference for concordance and continuity of care. Spearman correlation coefficients were used to assess correlations between (a) scores on each scale, (b) importance of and preference for concordance and continuity, and (c) demographic characteristics including age, marital status, education level, and household income. Modified Poisson regression with a robust error variance was used to calculate risk ratios (RR)^34^ and 95% confidence intervals (CI). We estimate the bivariate relationship between (a) demographic variables and heightened perceived racism, discrimination, and stress and subsequently (b) heightened perceived racism, discrimination, and stress and patient-reported importance of and preference for racial concordance with obstetric providers. All models were unadjusted. Among those who responded to the surveys, there were minimal missing data as all questions were required; three participants entered their date of birth incorrectly so were excluded from analyses requiring age. To address potential bias from lost-to-follow-up, two-sample t-tests and chi-squared tests were used to assess for differences in demographics between the initial and follow-up samples. Inverse probability censoring weights were constructed based on the significant predictors of lost-to-follow-up and applied to all analyses as a sensitivity analysis.^35^ All analyses were performed using STATA version 16 (College Station, TX). *p* Values < 0.05 were considered statistically significant.

## Results

Two hundred individuals consented to and completed the initial survey. The average number of children was 2.1 (SD = 1.4) and the average household size was 3.9 (SD = 1.3). The majority of participants were <35 years old (72.1%), non-Hispanic (*n* = 187, 93.5%), married (*n* = 104, 52.0%), reported household income under $50,000 annually (*n* = 107, 53.5%), and had at least a bachelor’s degree (*n* = 109, 54.5%) (Table 1). Sixty-nine participants completed the follow-up survey (34.5% of the total study population) and they were significantly more likely than the total population to be aged 35 or older (42.7% vs. 27.9%; *p* = 0.001), have a household income ≥$50,000K (62.3% vs. 46.5%; *p* = 0.0234), and marginally more likely to have a bachelor’s degree or higher (66.7% vs. 54.5%; *p* = 0.0778; Table 1).

**Table 1.** Participant Characteristics

	Initial survey (*n* = 200)		Follow-Up survey (*n* = 69)		
Characteristic	Mean	SD^a^	Mean	SD^a^	*p* Value^b^
Number of children	2.1	1.4	2.0	1.1	0.5957
Household size	3.9	1.3	4.0	1.1	0.3673
** **	**N**	**%**	**N**	**%**	***p* Value^c^**
Age					0.001
<35 years old	142	72.1	39	57.4	
≥35 years old	55	27.9	29	42.7	
Ethnicity					0.6126
Hispanic	6	3.0	3	4.4	
Non-Hispanic	187	93.5	65	94.2	
Chose not to answer	7	3.5	1	1.5	
Marital status					0.4712
Married	104	52.0	39	56.5	
Unmarried	97	48.5	30	43.5	
Education					0.0778
Less than bachelor’s degree	91	45.5	23	33.3	
Bachelor’s degree or higher	109	54.5	46	66.7	
Annual household income					0.0234
<$50,000	107	53.5	26	37.7	
≥$50,000	93	46.5	43	62.3	

^a^ Standard deviation.

^b^ Based on two-sample t-tests.

^c^ Based on chi-squared tests.

**FIG. 1.**
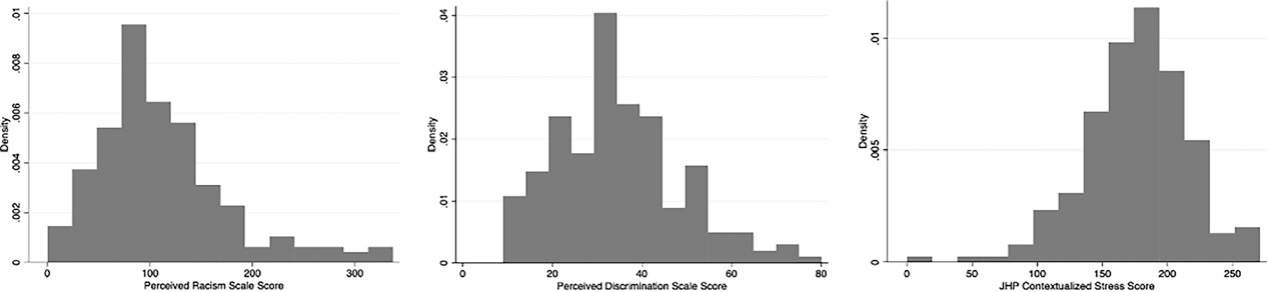
Distribution of Perceived Racism Scale, Perceived Discrimination Scale, and JHP Contextualized Stress Scale Scores. JHP, Jackson, Hogue, Phillips.

Among these 200 respondents, participants scored a median of 99.5 (total range 0–337, IQR 71–137.5) on the racism scale, 33 (total range 9–80, IQR 24.5–42) on the discrimination scale, and 177 (total range 0–271, IQR 153–203) on the contextualized stress scale (Fig. 1). There was a positive and statistically significant correlation between racism, discrimination, and contextualized stress. Racism and discrimination correlated most strongly with each other (r = 0.661, *p* < 0.001) (Table 2). In addition, racism and discrimination correlated with contextualized stress scores (r = 0.543, *p* < 0.001 and r = 0.520, *p* < 0.001, respectively). Increasing household income was negatively correlated with discrimination (r = −0.166, *p* = 0.019).

**Table 2.** Correlation Between Scales, Demographic Variables, and Concordance and Continuity Responses

	Racism	Discrimination	Contextualized stress	Marital status	Age	Education	Household income	Race importance	Gender importance	Continuity importance	Race preference	Gender preference	Continuity preference
Racism	1.000												
Discrimination	**0.661**	1.000											
Contextualized	**0.543**	**0.520**	1.000										
Marital status^b^	−0.050	−0.109	−0.027	1.000									
Age	0.052	−0.021	0.120	**0.268**	1.000								
Education^c^	0.046	−0.086	−0.010	**0.305**	**0.386**	1.000							
Household income^d^	−0.025	**−0.166**	−0.081	**0.474**	**0.333**	**0.664**	1.000						
Race importance	0.149	**0.325**	0.207	0.077	0.103	−0.028	0.136	1.000					
Gender importance	0.101	0.170	**0.263**	−0.172	0.086	−0.016	−0.076	**0.289**	1.000				
Continuity importance	0.148	0.103	−0.108	−0.166	−0.035	0.009	−0.105	0.118	0.197	1.000			
Race preference	0.145	0.186	0.149	0.067	0.040	0.088	0.168	**0.746**	**0.394**	0.096	1.000		
Gender preference	−0.082	−0.118	−0.001	0.073	0.097	0.161	0.042	0.171	**0.646**	0.192	**0.444**	1.000	
Continuity preference	0.092	0.179	−0.005	−0.123	0.082	0.201	−0.008	**0.296**	**0.409**	**0.438**	**0.370**	**0.464**	1.000

Bolded correlation coefficients indicate *p* < 0.05.

*n* = 200 for all correlations between scales and demographic variables; *n* = 69 for all correlations involving concordance or continuity.

^a^ All measured with Spearman correlation coefficients.

^b^ Marital status: unmarried = 0, married = 1.

^c^ Education: 0 = less than high school and 8 = doctorate.

^d^ Household income: 1 = <$25,000 annually, 7 = >$100,000 annually.

Of the 69 participants who completed both surveys, 78.3% either agreed or strongly agreed they would choose a racially concordant prenatal provider if given the opportunity (*n* = 54). In addition, 95.7% agreed or strongly agreed they would choose to have continuity with their prenatal provider if given the opportunity and 75.4% agreed or strongly agreed they would choose a gender-concordant provider if given the opportunity (*n* = 66 and *n* = 52, respectively). Scoring higher on the discrimination scale was positively correlated with agreeing that racial concordance with one’s prenatal provider is important (r = 0.325, *p* = 0.007) and scoring higher on the contextualized stress scale was positively correlated with agreeing that gender concordance with one’s prenatal provider is important (r = 0.263, *p* = 0.029) (Table 2).

In bivariate regression models, being unmarried was associated with double the risk of scoring in the top quartile of perceived discrimination (RR 2.06, 95% CI: 1.26–3.40) (Table 3). Age of 35 years or older was associated with 1.6 times the risk of scoring in the top quartile on contextualized stress (RR 1.61, 95% CI: 1.01–2.57) (Table 3). Annual household income under $50,000 annually was associated with significant risk of scoring in the top quartile of racism and discrimination (RR 1.85, 95% CI: 1.09–3.13 and RR 2.20, 95% CI: 1.30–3.74, respectively) (Table 3).

**Table 3.** Bivariate Associations Between Demographic Characteristics and Outcomes of Interest

	Scoring in the top quartile on the…						Agreeing or strongly agreeing with…			
	Perceived racism scale (*n* = 200)		Perceived discrimination scale (*n* = 200)		JHP^a^ contextualized stress scale (*n* = 200)		It is important for my prenatal provider to have the same race as me. (*n* = 69)		If given the opportunity, I would choose a prenatal provider with the same race as me. (*n* = 69)	
Demographic characteristic	RR^b^	95% CI^c^	RR^b^	95% CI^c^	RR^b^	95% CI^c^	RR^b^	95% CI^c^	RR^b^	95% CI^c^
Unmarried	1.25	0.77–2.02	**2.06**	**1.26–3.40**	1.34	0.83–2.15	0.92	0.52–1.62	0.89	0.69–1.16
Age 35 years or older	0.91	0.52–1.57	0.93	0.55–1.57	**1.61**	**1.01–2.57**	1.01	0.57–1.80	0.92	0.72–1.19
Education < bachelor’s degree	1.52	0.94–2.48	1.34	0.84–2.13	1.20	0.75–1.91	0.76	0.40–1.46	0.92	0.69–1.22
Household income < $50K/year	**1.85**	**1.09–3.13**	**2.20**	**1.30–3.74**	1.64	1.00–2.71	0.87	0.48–1.58	0.90	0.68–1.18

Bolded RRs indicate statistical significance.

^a^ JHP, Jackson, Hogue, Phillips.

^b^ RR, relative risk (unadjusted).

^c^ CI, confidence interval.

We found no significant associations between participant demographics and agreeing that racial concordance with one’s prenatal provider is important or preferable (Table 3). We also found no significant associations between reporting heightened experiences of racism, discrimination, and stress and agreeing that racial concordance with one’s prenatal provider is important or preferable (Table 4).

**Table 4.** Bivariate Associations Between Racism, Discrimination, and Stress Scales and Agreeing or Strongly Agreeing with Racial Concordance Statements

	It is important for my prenatal provider to have the same race as me. (*n* = 69)		If given the opportunity, I would choose a prenatal provider with the same race as me. (*n* = 69)	
Scoring in the top quartile on the …	RR^a^	95% CI^b^	RR^a^	95% CI^b^
Perceived Racism Scale	1.39	0.79–2.42	1.21	0.96–1.52
Perceived Discrimination Scale	1.08	0.58–2.00	1.09	0.84–1.41
JHP^c^ Contextualized Stress Scale	1.39	0.79–2.42	0.92	0.68–1.25

Bolded RRs indicate statistical significance.

^a^ RR, relative risk (unadjusted).

^b^ CI, confidence interval.

^c^ JHP, Jackson, Hogue, Phillips.

Sensitivity analyses upweighting underrepresented individuals in the follow-up survey (younger, lower education, and lower income) showed largely consistent findings, indicating minimal selection bias (Supplementary Appendix S2). Notably, we observed stronger relationships between household income under $50,000 with racism (RR 2.00, 95% CI: 1.17–3.44) and discrimination (RR 2.40, 95% CI: 1.39–4.15).

## Discussion

The majority of Black birthing people in our study experience significant racism, discrimination, and contextualized stress in their lives and nearly 80% would choose a racially concordant prenatal provider if given the opportunity. These findings parallel prior work by Mehra et al., Chambers et al., and Jackson et al. showing birthing people report significant exposure to racism, discrimination, and stress, as well as work by Altman et al., in which Black women recommended increasing racial concordance to improve pregnancy care.^28,36–38^

Consistent with our hypothesis, Black birthing people’s experiences of high levels of discrimination were positively correlated with agreeing that racial concordance is important. However, in regression analysis, no significant associations were found between the experience of discrimination, racism, and stress and agreeing racial concordance is important or preferable. We suspect these findings are due to the large proportion of study participants who reported that they would choose a racially concordant prenatal provider if given the opportunity. These feelings may be regardless of the amount of discrimination, racism, and stress they have experienced throughout their lives. These findings are consistent with prior studies exploring preference for racial concordance with prenatal providers.^25,26,28^ Future research is needed to investigate additional predictors of health care-based discrimination as well as methods for reduction of these experiences and their health impacts.^39^

We consider our findings assessing the relationship between demographic characteristics and outcomes with an intersectional lens.^8,9^ Intersectionality denotes that social categories interact, through which systems of oppression overlap and compound to perpetuate inequalities. In addition to race- and gender-based discrimination, previous studies have shown that marital status, age, and income may influence one’s experiences of discrimination in reproductive health settings.^40,41^ Our findings support this notion: in addition to being Black and birthing people, individuals who were unmarried, older, or of low income were significantly more likely to report racism, discrimination, and stress. However, they were no more likely than their counterparts to report importance or preference for racial concordance with providers. This discrepancy may be related to perceived access to such concordance, priority for other situations such as continuity of care, or a preference for concordance on another identity (e.g., age). While the current findings allude to the nuanced and very personalized experience of racism related to being not only a Black birthing person, but also single, older, or a low-income Black birthing person, they are only bivariate associations and make a call for further research in this space explicitly measuring the relationship between intersecting identities, racism, and identity concordance with providers.

Unfortunately in light of these findings, in 2021, only 10.7% of obstetrician/gynecologists and 7.3% of certified nurse midwives in the United States identified as Black, while 14.6% of birthing people did.^42–45^ In addition, the proportion of Black obstetrics/gynecology residents is decreasing.^44^ This explains why White birthing people are more likely to have racial concordance with their provider and Black birthing people are not. Given the fact that the existing research suggests that Black birthing people prefer racial concordance with their prenatal providers and that it is associated with higher levels of patient satisfaction, it is clearly evident that we need more Black-identifying obstetricians and nurse-midwives to provide care to the communities they serve. Given that Black birthing people experience the highest levels of perinatal morbidity and mortality than people of any other race, improving obstetric care for Black birthing people should be a top priority for all stakeholders in this space. One way to do that is to develop policies and programs that increase the number of Black-identifying individuals in the medical field.

Of note, nearly 80% of study participants reported that they would choose to have continuity with their prenatal provider if given the opportunity, implying that continuity of prenatal provider is a high priority for Black birthing people. Having continuity with one’s prenatal provider may allow for relationship building, trust, and inclusive communication and decrease the need to explain their stressors to various providers across their prenatal course. Prior studies have indicated these are important factors in quality prenatal care.^26,28,46^

We are the first study to examine racism, discrimination, and contextualized stress in relation to Black birthing peoples’ perspectives regarding the importance of racial concordance and continuity with their prenatal provider. An additional strength is the intentional focus on Black birthing people and elevation of their lived experience. We used validated scales and, where applicable, measures specifically designed for Black women (contextualized stress scale) to assess these experiences. Lastly, we directly assessed if participants would choose a racially concordant provider if that was an option, which allows us to draw meaningful conclusions about how to change prenatal care provision to align with Black birthing patient’s values.

The primary limitation of this study is the follow-up survey’s response rate, which resulted in small sample sizes and a decreased ability to detect associations between racism, discrimination, and stress with desires for racial concordance. Despite small sample sizes, positivity was not an issue in the bivariate models, which had at least 5 observations in every cell. Relatedly, models were unadjusted and may suffer from confounding; regression estimates should be interpreted as crude, bivariate measures of association. Furthermore, those who responded to the follow-up survey differed from those who answered the initial survey in terms of marital status, education, and income. However, sensitivity analyses upweighting underrepresented individuals in the follow-up survey showed minimal evidence of selection bias.^35^ Referral bias may also be present because all participants received care within the university health care system and therefore may have had higher acuity in their pregnancy, which may be associated with higher levels of stress and psychological distress.

## Health Equity Implications

Black birthing people experience significant racism, discrimination, and contextualized stress and the majority would prefer racial concordance and continuity of care with their prenatal provider. These findings contribute substantively to the emerging literature of research exploring racial concordance between prenatal providers and Black birthing people. Solving the maternal health equity crisis in the United States is going to require a myriad of strategies and enormous structural change. One small step in the right direction, however, may be to heed the findings of this study and create the opportunity for Black birthing people to have a racially concordant prenatal provider with whom they can have continuity should they so choose. In the meantime, efforts must be enacted to improve cultural competency among existing providers to facilitate relationships between nonconcordant birthing people and their providers that engender trust, safety, and respect.

## Abbreviations Used

CI: Confidence interval
IQR: Interquartile range
JHP: Jackson, Hogue, Phillips
REDCap: Research Electronic Data Capture
RR: Risk ratios
