# Supplementary material for: Examining Black Birthing People’s Experiences with Racism, Discrimination, and Contextualized Stress and Their Perspectives on Racial Concordance with Prenatal Providers
Source: Health Equity. 2024 Sep 12;8(1):588–98. doi: 10.1089/heq.2023.0266 (PMC11464861; doi:10.1089/heq.2023.0266)
Supplement: Supplementary Appendix S1 [file heq.2023.0266_suppl_datas1.pdf]

Appendix I. Demographic Questionnaire and Follow-Up Survey

Demographic Questionnaire

1. What is your age in years?
2. What is your race? Select all that apply.
   - Black or African American
   - White or Caucasian
   - American Indian or Alaskan Native
   - Asian
   - Native Hawaiian or Pacific Islander
   - Other: _________
3. What is your ethnicity?
   - Hispanic
   - Non-Hispanic
4. What is your marital status?
   - Married
   - Divorced
   - Separated
   - Single
   - Cohabitating
   - Widowed
5. What is your highest degree of education? (If you are currently in school, please list the highest completed degree to date.)
   - Less than high school diploma
   - High School Degree
   - Some college, no degree
   - Associates Degree
   - Bachelor's Degree
   - Master's Degree
   - Professional Degree
   - Doctorate
6. What is your household income?
   - Less than 20,000
   - 20,000-34,999
   - 35,000-49,999
   - 50,000-64,999
   - 65,000-79,999
   - 80,000-99,999
   - > 100,000
7. How many times have you been pregnant?
8. How many children do you have?
9. Have you ever had a preterm delivery (baby born before 37 weeks)?
   If yes, how many weeks pregnant were you when you delivered?
10. What is the month and day of your last delivery?
11. What kind of insurance did you have at the time of your last delivery?
    - None
    - Medicaid
    - Private Insurance
12. Please check the box if you have the following medical conditions. Select all that apply.
    - Chronic hypertension (High blood pressure)
    - Hypertension caused by pregnancy
    - Gestational Diabetes or diabetes caused by pregnancy (controlled with diet)
    - Gestational Diabetes or diabetes caused by pregnancy (controlled with medication)
    - Type 2 Diabetes
    - Type 1 Diabetes
    - Substance use or abuse
    - Anxiety
    - Depression
    - Bipolar Disorder
    - Other: _____________

Follow-Up Survey

1. It is important for my obstetric provider to have the same race as me.
    - Strongly agree
    - Agree
    - Neutral
    - Disagree
    - Strongly disagree

2. It is important for my obstetric provider to have the same gender as me.
    - Strongly agree
    - Agree
    - Neutral
    - Disagree
    - Strongly disagree

3. It is important for me to see the same obstetric provider for all of my prenatal visits.
    - Strongly agree
    - Agree
    - Neutral
    - Disagree
    - Strongly disagree

4. If given the opportunity, I would choose an obstetric provider with the same **race** as me.
    - Strongly agree
    - Agree
    - Neutral
    - Disagree
    - Strongly disagree

5. If given the opportunity, I would choose an obstetric provider with the same **gender** as me.
    - Strongly agree
    - Agree
    - Neutral
    - Disagree
    - Strongly disagree

6. If given the opportunity, I would choose to see the **same obstetric provider for all of my prenatal visits**.
    - Strongly agree
    - Agree
    - Neutral
    - Disagree
    - Strongly disagree

7. As you reflect on race, gender, and having the same obstetric provider for all prenatal visits , please rank them in the order of importance to you with #1 being the most important.
    - Race
    - Gender
    - Having the same obstetric provider for all prenatal visits
