# Supplementary material for: Examining Black Birthing People’s Experiences with Racism, Discrimination, and Contextualized Stress and Their Perspectives on Racial Concordance with Prenatal Providers
Source: Health Equity. 2024 Sep 12;8(1):588–98. doi: 10.1089/heq.2023.0266 (PMC11464861; doi:10.1089/heq.2023.0266)
Supplement: Supplementary Appendix S2 [file heq.2023.0266_suppl_datas2.pdf]

Appendix II. Sensitivity analysis using IPCWs on associations between demographic characteristics and outcomes of interest

	Scoring in the top quartile on the…						Agreeing or strongly agreeing with…			
	Perceived Racism Scale		Perceived Discrimination Scale		JHP Contextualized Stress Scale		It is important for my prenatal provider to have the same race as me.		If given the opportunity, I would choose a prenatal provider with the same race as me.	
Demographic Characteristic	wRR	95% CI	wRR	95% CI	wRR	95% CI	wRR	95% CI	wRR	95% CI
Unmarried	1.32	0.80 - 2.19	**2.07**	**1.22 - 3.51**	1.31	0.79 – 2.19	0.84	0.44 - 1.60	0.84	0.64- 1.09
Age 35 years or older	0.82	0.47 - 1.43	0.84	0.50 - 1.43	**1.68**	**1.07 – 2.66**	1.11	0.60 – 2.03	0.90	0.68 - 1.19
Education < bachelor's degree	1.60	0.97 - 2.66	1.28	0.80 - 2.05	1.18	0.72 - 1.94	0.85	0.43 – 1.69	0.92	0.70- 1.20
Household income < $50K/year	**2.00**	**1.17 - 3.44**	**2.40**	**1.39 – 4.15**	1.54	0.92 - 2.59	0.74	0.38 – 1.43	0.96	0.74 - 1.24

Bolded RRs indicate statistical significance
IPCW: Inverse Probability of Censoring Weight; JHP: Jackson, Hogue, Phillips; wRR: Weighted Relative Risk; CI: Confidence interval

Appendix III. Sensitivity analysis using IPCWs on association between racism, discrimination, and stress scales and agreeing or strongly agreeing with racial concordance statements

	It is important for my prenatal provider to have the same race as me.		If given the opportunity, I would choose a prenatal provider with the same race as me.	
Scored in the top quartile of the…	wRR	95% CI	wRR	95% CI
Perceived Racism Scale	1.42	0.74 – 2.71	1.15	0.88- 1.48
Perceived Discrimination Scale	1.01	0.49 - 2.07	1.01	0.4 - 1.37
JHP Contextualized Stress Scale	1.38	0.73 - 2.63	0.88	0.3 - 1.23

Bolded RRs indicate statistical significance
IPCW: Inverse Probability of Censoring Weight; wRR: Weighted Relative Risk; CI: Confidence interval; JHP: Jackson, Hogue, Phillips
